# Knockdown of Dinoflagellate Condensin *CcSMC4* Subunit Leads to S-Phase Impediment and Decompaction of Liquid Crystalline Chromosomes

**DOI:** 10.3390/microorganisms8040565

**Published:** 2020-04-14

**Authors:** Ting Hin Kosmo Yan, Zhihao Wu, Alvin Chun Man Kwok, Joseph Tin Yum Wong

**Affiliations:** Division of Life Science, Hong Kong University of Science & Technology, Clear Water Bay, Kowloon, Hong Kong, China; Kosmo@connect.ust.hk (T.H.K.Y.); wuzhihao@hotmail.com (Z.W.); alvink@ust.hk (A.C.M.K.)

**Keywords:** condensin, dinoflagellate, CcSMC4, cell cycle, liquid crystalline chromosomes, S phase, chromosome compaction

## Abstract

Dinoflagellates have some of the largest genomes, and their liquid-crystalline chromosomes (LCCs) have high degrees of non-nucleosomal superhelicity with cation-mediated DNA condensation. It is currently unknown if condensins, pentameric protein complexes containing structural maintenance of chromosomes 2/4, commonly involved in eukaryotic chromosomes condensation in preparation for M phase, may be involved in the LCC structure. We find that CcSMC4p (dinoflagellate SMC4 homolog) level peaked at S/G2 phase, even though LCCs do not undergo global-decondensation for replication. Despite the differences in the chromosomal packaging system, heterologous CcSMC4p expression suppressed conditional lethality of the corresponding fission yeast mutant, suggesting conservation of some canonical condensin functions. CcSMC4p-knockdown led to sustained expression of the S-phase marker PCNAp, S-phase impediment, and distorted nuclei in the early stage of CcSMC4p depletion. Prolonged CcSMC4p-knockdown resulted in aneuploidal cells and nuclear swelling with increasing LCC decompaction–decondensation. Cumulatively, our data suggested CcSMC4p function was required for dinoflagellate S-phase progression, and we propose that condensin-mediated higher-order compaction provisioning is involved in the provision of local rigidity for the replisome.

## 1. Introduction

Accumulating evidence suggests chromatin remodeling between interphase and mitosis goes through condensin-dependent stages to their higher-order organization of nucleosomal chromosomes [1]. Condensin dysfunction leads to phenotypes depending on the chromosomal interactive point of the cell cycle. Condensin structural maintenance of chromosomes 2 and 4 subunits (*SMC2* and *SMC4*) are essential genes in nucleosomal eukaryotes. 

Dinoflagellate liquid crystalline chromosomes (LCCs) are the only non-nucleosomal eukaryotic chromosome condensation-organization; despite having some of the largest genome sizes, micrococcal nuclease-digested LCC preparations do not exhibit a nucleosomal ladder pattern [2,3,4]. Ultrastructural chromosome spread studies have revealed a high level of DNA superhelicity [5]. Strong birefringence and re-interpreted biophysical data support LCCs having liquid crystalline anisotropic organization in vivo [6,7]. Divalent cation-mediated condensation and DNA supercoils are synergistic in nurturing DNA liquid-crystalline phase transitions [8], and LCCs have 2–3 fold higher chromosomal divalent cations when compared to nc-chromosomes [9]. Cation chelation led to the orchestrated remodeling of higher-order structures, contrasted with little changes in nucleosomal chromatin following similar treatment [10,11,12]. LCCs recompacted upon re-introduction of divalent cations after mild chelation [12], implicating cation-mediated superhelical subunit modularity [12,13].

Condensins are pentameric protein complexes composed of SMC2p (*cut 14* is fission yeast homologue) and SMC4p (cut 3p) core subunits, and three non-SMC proteins (CapHp/cnd2p, CAP-D2p/cnd1p, and CAP-Gp/cnd3p) [14]. Each SMC protein has two terminal Walker domains, a central Hinge segment, and the long helix-loop-helix coiled-coils in between; SMC2 and SMC4 subunits fold back at central hinge domains and hetero-dimerize between their n- and c-terminal Walker domains, forming two head ATPase domains. In vivo degron experiments have suggested chromatins are restraint between long SMC2/4 heterodimeric helix–loop–helix domains, which form structures referred to as the condensin “rings” [15]. The Head domains bind to the three non-SMC proteins, chromosome associated protein H (CapH*^cnd2^*) and the two heat repeat-motif-containing proteins CAP-D2*^cnd1^* and CAP-G*^cnd3^*, forming a compressible belt around chromatin domains [16,17,18]. There is much controversy regarding condensin molecular mechanisms of action, with an ongoing model involving DNA loop extrusion with condensin sliding [19,20], in addition to the linkage of condensin-entrapped domains [1]. Bacterial SMC homologs (*MukBEF*, *MukB*) organized nucleoid DNA into a repetitive stable structure [21,22].

Predicted polypeptides of CcSMC4p and CcSMC2p (from *Crypthecodinium cohnii* transcriptomes) contained all conserved domains of eukaryote SMC homologs (Figure S1), including the N- and C-terminal ATPase walker motifs and the central hinge region. Higher levels of chromosomal cations and cation-aided liquid-crystalline phase transition might have made condensins redundant. However, the high level of superhelicity and the absence of nucleosomal architecture inspired us to pioneer this functional investigation of a dinoflagellate chromosomal protein. We adopted multiple approaches to investigate possible functions of condensin subunit CcSMC4p, including the first gene-knockdown of a dinoflagellate chromosomal protein. Our data implicated dinoflagellate condensins being required for proper S-phase progression, likely related to their provision of replisome organizational rigidity. 

## 2. Materials and Methods

### 2.1. Dinoflagellate Cell Cultures and Flow Cytometry 

The heterotrophic dinoflagellate *Crypthecodinium cohnii* (University of Texas Culture Collection strain 1649) was maintained in the MLH minimum medium without light at 28 °C (Tuttle and Loeblich III, 1975). Cells for flow cytometry were prepared as previously described [23]. Knockdown experiments and cell-cycle studies were conducted with *C. cohnii* cells. There are established transfection and coccoid stage-swarmer release synchronization protocol [24,25]. 

### 2.2. In Silico Analysis 

ORFs encoding CcSMC2 and CcSMC4 (*Crypthecodiunium cohnii*) were originally cloned by sequence homology and subsequently confirmed with contigs constructed with expressed sequence tags from transcriptomes. The predicted amino acid sequences of dinoflagellate CcSMC2 and CcSMC4 ORFs were compared with other eukaryotic homologs from selected eukaryotic homologs (Figure S1) using multiple alignment tools (https://www.ebi.ac.uk/Tools/msa/clustalo/). GenBank accession number: KC160503 for *CcSMC2* and KC160504 for *CcSMC4*.

### 2.3. Immunological Techniques, Preparation of Cell Lysates and Molecular Biological Techniques

Purified bacterial expressed recombinant polypeptides (His-tagged, vector PQE30, Qiagen) containing *N*-terminal coiled-coil region (predicted 369th–769th amino acids of CcSMC4p) and *C*-terminal coiled-coil region (predicted 895th–1323rd amino acids of CcSMC4p) were the immunogens for the anti-CcSMC4p antibody. Baculoviral-expressed (Thermal Fisher Scientific) recombinant polypeptides encoding full-length CcSMC2p were the immunogens for the anti-CcSMC2p antibody. All immunological manipulations were carried out according to the published protocols [23,26]. The affinity-purified antibodies against CcSMC4p or CcSMC2p recognized immunoreactive bands with approximate expected molecular weights in *C. cohnii* cell lysates (~170 kDa and ~137 kDa, respectively; Figure S2B). Antigen affinity-purified antibodies were used in all immunological techniques; all cell lysate blots were pre-cleared with dried acetone bacteria extract before immunoblot analysis [23,25,27]. 

All fluorescent photomicrographs were taken with a Leica fluorescent microscope (DMLS), and confocal images were taken with Leica SP8 (Leica microsystems, Witzlar, Germany). All molecular biology and protein preparation techniques adopted our previously published protocols, including the preparation of cell lysates [23], which was based on a pressure-release cell disruption method (Cell Disruptor, Constant System, UK). Dinoflagellate proliferative cell nuclear antigen (PCNA), which had expression peak at S-phase [28,29], was the S-phase marker. The anti-PCNAp (PC10) and anti-α-tubulin monoclonal antibodies were from ZyMed Corporation (San Francisco, CA, USA). All chemicals were from Sigma Aldrich unless otherwise stated.

### 2.4. Functional Suppression of cut3-477 Conditional Lethality through CcSMC4p Expression 

Potential CcSMC4p-mediated suppression of conditional lethality of *cut3-477,* a temperature-sensitive fission yeast (*Schizosaccharomyces pombe*) mutant in SMC4 homolog [30,31], was conducted for functional studies. Fission yeast strains and expression vectors were gifts from Prof. Paul Nurse (Oxford University) and Prof. Mitsuhiro Yanagida (Okinawa Institute of Science and Technology, Okinawa, Japan). The vector expressing CcSMC4 open reading frame, driven by the thiamine-repressible *nmt1* promoter, was constructed in the Rep3x vector [32] and transformed into fission yeast mutants under unrepressed (0 µM thiamine), fully-repressed (15 µM thiamine), or semi-repressed (2.5 µM thiamine) conditions. Fission yeast cell lysates were prepared from transformed cells incubated in supplemented Edinburgh minimal media (EMM )with or without thiamine (15 µM) for 24 h at 28 °C. All yeast manipulation and protein preparations followed established protocols [33].

### 2.5. Antisense Oligonucleotide-Mediated Gene-Knockdown Experiment

We adopted an antisense-oligonucleotide based gene-knockdown protocol [27] with lipofection-spheroplasts mediated transfection in *Crypthecodinium cohnii* [25,34], according to manufacturer protocol (Lipofectamine, Invitrogen). The design of antisense oligonucleotides was aided with unpaired bases predicted on *CcSMC4* mRNA (http://rtools.cbrc.jp/centroidhomfold/) [35]. Transfecting a combination of two anti-sense ODNs (SMC4-79, 5′-AAGGTGCGGTCAGGTGGAAACC and SMC4-129, 5′-TAGATCGGGTTGTGACGGGCATGAC) gave the best knockdown reduction of CcSMC4p (Figure S2). Control-ODNs, encoding the sense (complementary) sequence of the SMC4-79 oligo, were used as a control in the mock-transfection. Many metazoans have two condensin complexes, differing in their heat-repeat subunits and chromosome functions [36], our antisense-oligonucleotide design would have targeted both complexes as the SMC proteins are shared. All experiments were carried out in triplicates, and the representative results are presented. 

## 3. Results 

### 3.1. Cell-Cycle-Phased Expression of CcSMC4p 

**Figure 1:**
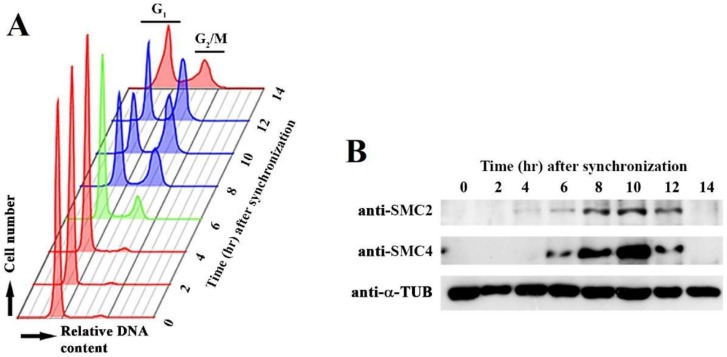
CcSMC4p (dinoflagellate SMC4 homolog) expression in the cell cycle. (**A**) Flow cytograms of *Crypthecodinium cohnii* cells collected at different time points after synchronization at early G_1_. Red, G_1_; Green, S phase; purple G_2_/M cells. (**B**) Immunoblots of CcSMC2 and CcSMC4 collected at the corresponding time points.

Condensin functions are regulated in the cell cycle. We followed CcSMC4p and CcSMC2p levels in *C. cohnii* cell cycle with immunoblot analysis (Figure 1). Despite no apparent LCC decompaction–recompaction cycles in dinoflagellates, low apparent early G_1_ level (T = 0–4 hr) was elevated at S phase (T = 5–6 hr), before leveling at the G_2_/M (T = 8–12 hr) (Figure 1). This S-G_2_ expression pattern implicated possible cell-cycle operations for both SMC homologs; in nucleosomal eukaryotes, the SMC2/SMC4 levels did not change appreciably during the cell cycle [34]. Further studies should investigate possible post-translational modifications and subcellular locations of condensins. 

### 3.2. Heterologous CcSMC4p Expression Suppressed Conditional Lethality of Fission Yeast cut3-477

Suppression of conditional lethality in their corresponding yeast mutants would be a good proof of functional conservation. In addition to substantial evolutionary distances between yeast and dinoflagellates, the heterologous CcSMC4p subunits need to form functional complexes with the yeast SMC2*^cut3^* and non-SMC subunits. This may be too severe a test, but we were hopeful CcSMC4p might at least partially suppress the *cut* phenotype in the corresponding fission yeast temperature-sensitive mutant *cut3-477* [31].

**Figure 2:**
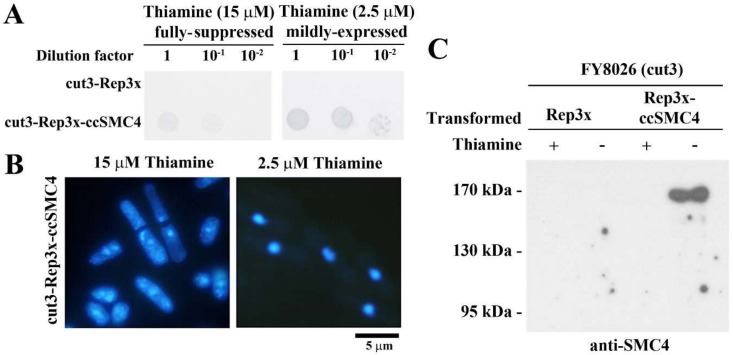
CcSMC4p expression suppressed conditional lethality of fission yeast *cut3-477.* (**A**) Spot-dilution assay demonstrating functional suppression of conditional lethality in *cut3-477* with CcSMC4 expression driven by thiamine-repressible nmt1 promotor [32]. Serial dilutions of overnight transformant cultures were spotted on EMM agar, supplemented with thiamine at fully repressed (15 µM) or partially repressed (2.5 µM) concentrations and incubated at a restrictive temperature of 36 °C for 3 days. (**B**) Fluorescent photomicrographs of DAPI-stained CcSMC4-transformed *cut3-477* cells at partially repressed (2.5 µM) and fully repressed conditions (15 µM). Arrows pointed to septation without completion of genome replication. (**C**) Immunoblot of cell lysates prepared from CcSMC4-transformed *cut3-477* cells with the anti-CcSMC4p antibody.

Unexpectedly, we observed suppression of *cut3-477* conditional lethality with CcSMC4p expression, at semi-repressive condition (2.5 µM thiamine and lesser so at 7.5 µM; Figure 2A,B). Fluorescent microscopy of DAPI-stained rescued *cut3-477* cells showed well-compacted nuclei with no *cut* phenotype at semi-repressed (2.5 µM thiamine) expression, but not under fully repressed condition (15 µM thiamine [32]; Figure 2B). This phenotypic suppression suggested CcSMC4p had the required canonical SMC4 functions and implicated interactions with the corresponding components in the yeast complexes. Immunoblot confirmed CcSMC4p was expressed in the transformed *cut3-477* cells (Figure 2C).

### 3.3. Prolong CcSMC4p-Knockdown Led to S-Phase Impediment with Nuclear Swelling 

**Figure 3:**
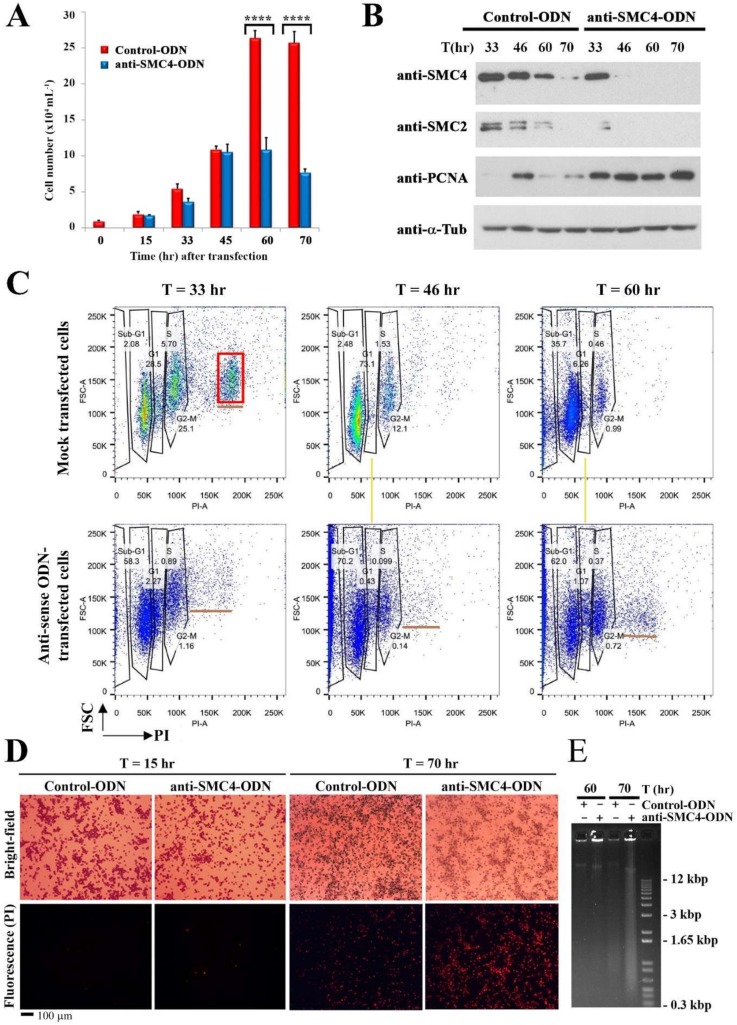
CcSMC4p-knockdown led to S-phase impediment. (**A**) Cell number of mock-transfected and ak-cells observed during the experiment from T = 0 to 70 h. Asynchronous cells were used for large-scale experiments. Data represent means ± standard error of three replicate experiments. Asterisk indicates a significant (*p* < 0.0001) difference compared with the control. (**B**) Immunoblot analysis for CcSMC4p and PCNAp in cell lysates prepared from later time points (T = 33, 45, 60, 70 h) in mock-transfected and ak-cells harvested at indicated time points post-transfection. Data for earlier time points are presented in Figure 5D. (**C**) DNA flow cytograms of cells collected from T = 33 to T = 60 h. *x*-axis: propidium iodide staining; *y*-axis: forward scatter. (**D**) Propidium iodide staining of control-ODN/anti-SMC4-ODN-transfected *C. cohnii* cells at T = 15 and 70 h post-transfection. (**E**) Ethidium bromide-stained agarose gel showing large molecular weight DNAs extracted from control-ODN and ak-cells harvested at T = 60 and 70 h post-transfection. Red box indicated multiple fission cells^39^. Yellow lines align anuploidal ak-cells with the S-phase cells in mock transfection.

Bearing in mind *SMC4*-knockouts commonly cause lethality, we adopted a gene-knockdown approach to study possible *CcSMC4* functions. Cell proliferation in antisense-ODN-treatment (ak-cells) dropped between 24–33 h when compared to the mock-transfected cells, which continued to proliferate during the experimental period (Figure 3A). This indicated the effect of CcSMC4p-knockdown being manifested early in the experiment and that most ak-cells had been transfected with the antisense-ODNs. Gene-knockdown would not have affected the pre-existing CcSMC4p, which in other systems have both cytoplasmic and nuclear populations [37]. Immunoblot suggested this CcSMC4p pool dropped to undetectable levels by T = 46–70 h(Figure 3B). However, there was a sustained level of the S-phase marker PCNAp, contrasted with the periodic expression in the mock-transfected cells (Figure 3B). A flow cytogram of ak-cells exhibited sustained sub-2N populations in all later samples, contrasted with the clear gaps between G_1_–G_2_ peaks peaks (yellow line, Figure 3C). There is no global decondensation during dinoflagellate DNA replication, a consequence of no normal S-phase bridge between G_1_–G_2_, and, hence, there are clear gaps between G_1_ and G_2_/M flow peaks [37,38].

**Figure 4:**
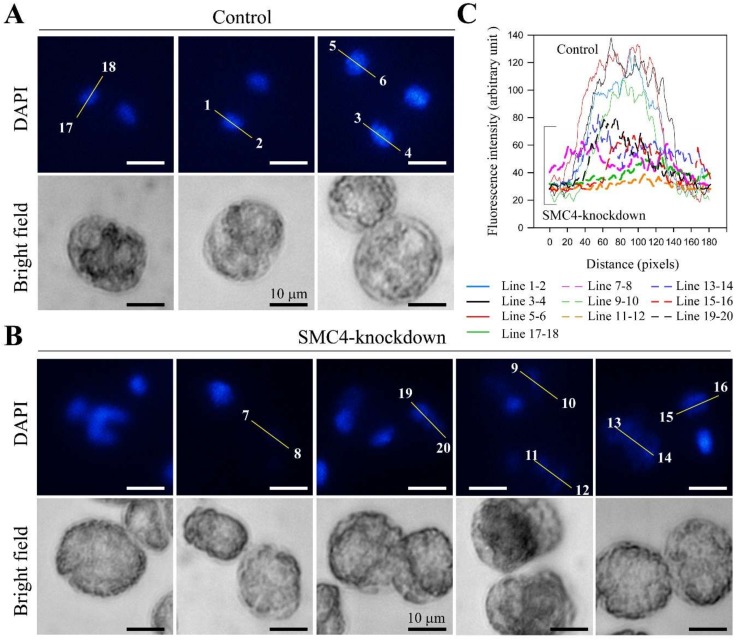
CcSMC4p-knockdown led to enlarged nuclei with reduction in DAPI staining. Fluorescent photomicrographs of (**A**) control cells (mock-transfected with sense-ODN); (**B**) ak-cells (T = 33 h) stained with DAPI; (**C**) quantification of fluorescent level along transects in (**A**) and (**B**). Numbers on the fluorescent photomicrographs indicate the directions and line numbers for each line transect. In CcSMC4p-knockdown cells, much lower levels and spread-out of DAPI-staining suggested enlarged nuclei when compared to control nuclei of mock-transfected cells; some ak-nuclei (e.g., 11–12, 13–14) were more enlarged than others (e.g., 15–16) when contrasted with condensed nuclei in ak-cells and totally decompacted LCCs after AMSA treatment (Figure S3B).

DNA-flow cytograms exhibited small cell populations with aneuploidy greater than the G2/M peak (Figure 4C, underlined; not 4N, red-lined for comparison). These cells were distinct from the normal clearly separated G_1_ and G_2_/M flow peaks, or the multiple fission cells (red square, T = 33 h), which is a special growth rate mediated divisional mode [39]. 

Transect analysis of the DAPI-stained image confirmed that many ak-nuclei (e.g., transect 11–14) increased in volume when compared to regular sizes in control nuclei (1–6, 17–18); all AK-nuclei had level DAPI lower than half that of control nuclei, with some cells losing DAPI stainability (transect 7–8; Figure 4B,C). There were nuclei with different degrees of DAPI-staining, some with distorted nuclei and some with hardly detectable staining. This was consistent with LCC decompaction in some ak-cells, as observed in chelation-mediated LCC decompaction in EDTA-treated nuclei [12]. As an independent control, we conducted a set of experiments with topoisomerase II inhibitor (AMSA) that induced *C. cohnii* LCC decondensation [37]. As in the case of some ak-cells (T = 33, transects 11–12,13–14, 19–20, and T = 70; Figure S3A), the diffused DAPI-staining (after AMSA treatment) of the whole swelled nucleus filled the whole cells (Figure S3B), suggesting decondensed LCCs resulting from CcSMC4p knockdown. These progression from decompacted to decondensed nuclei (T = 33–70 hr), and the aneuploidal profiles (Figure 3C) were consistent with the extended period of CcSMC4p-knockdown leading to increasing mis-accessibility to the pre-replicative complex [40], consistent with some ak-cells progressing to partial re-replication. Consistently, PI-staining suggested there were associated increases in cell deaths in latter timepoints associated with DNA breakage; the DNA streak (Figure 3E) LCC decondensation (Figure 4, Figure S3A) and the aneuploidal flow cytograms (Figure 3C) suggested they were not the results of apoptosis. Condensins are also involved in DNA damage repair responses [41,42] and increasing decompaction with condensin non-replacement will further aggravate replicative stress and amplify no-return effects on LCC decompaction-re-replication.

Continuous overlapping between 1N–2N DNA peaks in later timepoints, instead of clearly distinct G_1_ versus G_2_/M peaks in the ak-cells (yellow line), indicated DNA replication was impeded at intermediate stage. Multiple fission, having a 4N stage in addition to 1N and 2N, is a strictly growth-rate dependent mode of cell division in *C. cohnii* [39], represented as a distinct third peak (4N) in DNA flow cytograms of mock-transfected cells (red square box, Figure 3C, but not in ak-cells. Instead of this growth-dependent division mode, the aneuploidal sub-4N (b, underlined) population, towards the latter part of the experiment, was consistent with origin misfiring and semi re-replication in ak-cells. 

### 3.4. CcSMC4p-Knockdown Led to S-Phase Impediment from the First Cell Cycle

**Figure 5:**
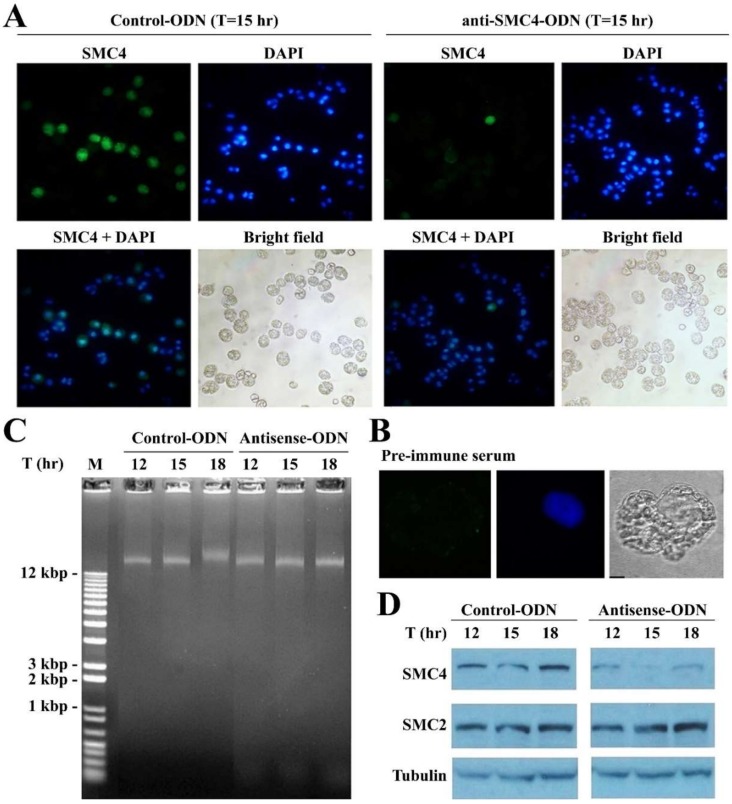
CcSMC4p-knockdown in synchronized cells led to S-phase impediment. (**A**) Fluorescent photomicrographs of CcSMC4p immunofluorescent signals and DAPI-staining in double-stained mock-transfected and ak-cells collected at T = 15 h post-transfection. Green immunofluorescent signals correspond to CcSMC4p. Blue fluorescent signals corresponded to DAPI-staining. CcSMC4p + DAPI: Merged CcSMC4p signals with DAPI signals. Not all cells were labelled strongly, which might be related to the highly compacted state of LCCs, and the use of affinity-purified antibody. (**B**) Immunofluorescent labeling with pre-immune serum. (**C**) Total DNA preparations from samples in (**A**) were analyzed with 1% agarose gel post-stained with ethidium bromide. Large molecular weight DNA with retarded relative mobility was indicative of replication intermediates [43]. (**D**) Immunoblot analysis of the mock-transfected and ak-cells collected at T = 12th, 15th, 18th hr after transfections for CcSMC4p, CcSMC2p, and α--tubulin.

These observations suggest that CcSMC4p functions are required for proper replisome progression. We thus sought independent evidence to verify if CcSMC4p-knockdown may affect the first cell cycle, as residue CcSMC4p might not be depleted to a critical level. In experiments using synchronized G_1_ cells for transfection, retardation of large molecular weight DNAs that is indicative of replication intermediates [43] was observed at T = 18 h, corresponding to the relative reduction in CcSMC4p in the mock-transfected cells, suggesting entry to S phase synchronously at between T = 15–18 h (Figure 5B). This did not occur in the ak-cells. CcSMC4p immuno-labeling in the mock-transfected cells (T = 15 h) labeled most cells strongly, with both cytoplasmic and overlapping distribution with DAPI-staining (Figure 3A). CcSMC4p levels in some ak-cells were undetectable, as in the immunoblot and with the pre-immune serum (Figure 5A,B). Most cells in both mock-transfected and ak-cells had condensed DAPI-staining, contrast with cells in later time points of ak-cells (Figure 4). As most cells did not enter the first S phase (cell proliferation halted between T = 24–33 h), this also registered the experimental timeframe, with apparent depletion of CcSMC4p by T = 33 h, to within one extended cell-cycle.

## 4. Discussion

No global decondensation occurs during dinoflagellate DNA replication, and LCCs only undergo local decompaction during S-phase. Nucleosomal chromatin rigidity is contributed by nucleosome–nucleosome interactions [44], reinforced by this condensin-mediated chromosome domain association linker [15,18,45,46,47]. Replisome maintenance involved strictly orchestrated nucleosome disassembly–reassembly; during chromosome replication, replisome components interacted with nucleosomal histones ahead of the replicon [15,48,49]. Condensin association was important for replication forks converging at rRNA loci and was proposed to have a role in other loci [50,51]. Loss of chromosome rigidity resulted in conditional mutants of fission yeast *cut3^ts^* and *cut14 ^ts^* [31] and the ring structures of the condensin complexes were important in the maintenance of chromosomal rigidity [15]. The increasing nuclear size and reduction in chromosome DAPI-staining (Figure 3A, Figure 5A, and Figure S3A) suggest that extended CcSMC4p-knockdown leads to increasing non-replacement of chromosomal condensins, consequenced with decompacted LCC domains with reduced rigidity. CcSMC4p-knockdown-mediated S-phase impediment implicated some condensin functions were required for proper S-phase progression. This implicates a role of condensin-mediated LCC compaction in providing replisome rigidity, failure of which would likely lead to replisome stalling and replicative stresses.

The degree of eventual decondensation was comparable to those observed in cation-chelated [12] or with topo II inhibitor-treated LCCs [37] (Figure S3B) in which swelled nuclei filled the size of a normal cell. This suggested that condensins were involved in LCC compaction, likely in the restraint of proposed superhelical modules [7]. Our working model rests with CcSMC4p-knockdown leading to replisome impediment in the early part of the experiment, and further replicative malfunctions with extended CcSMC4p depletion (Figure 5), contributed from origin mis-firings as a result of unwarranted accessibility of pre-replicative complexes [52] to cell cycle activators [53,54]. Premature replication termination led to nucleokinesis-cytokinesis as a result of licensing site exposures at un-recompacted replicons [55,56]. Condensins also participate in DNA damage responses, including single-strand DNA break repair [41,42], CcSMC4p-knockdown likely compromised the ability of DNA damage repair in ak-cells, contributing to sustained LCC lesions, further decompacted chromatins, and S-phase arrest. These were also manifested with increasing sub-G_1_ peaks, aneuploidal DNA peaks, and DNA breakages (T = 60, 70 h; Figure 3), as well as nuclear swelling with LCC decompaction (Figure 4B). Continued CcSMC4p-knockdown led to LCC decondensation in later time points (Figure S3A), in agreement with condensin-mediated compaction contributing to overall LCC compaction. As there was only local decompaction during S phase [51], LCC decondensation and S-phase impediment with sustained CcSMC4p-knockdown also implicated condensin loading per replication throughout the LCCs.

Although there is no information relating to replication termination mechanisms [40] in LCCs, continued origin mis-firing likely caused depletion of replication complex (e.g., cdc6, cdt1) from the nucleus and mis-signaling of replication termination to G_2_-coupled chromosome segregation. This is effectively the *cut* phenotype in which septation occurs without completing nucleokinesis [30,57,58]. Similarly, the *titan* seed mutant of *Arabidopsis ttn3* (*Titan* mutant of SMC2 homolog) was associated with a leaky phenotype of anaphase with remnant phragmoplasts and polyploid-like replication products [59]. Bacterial condensins and histone-like HU protein promote origin location and are required for genome segregation [60,61]. It is interesting that condensins are also required for liquid crystalline genomes.

## Supplementary Materials

The following are available online at https://www.mdpi.com/2076-2607/8/4/565/s1, Figure S1: Predicted amino acid sequences of dinoflagellate CcSMC2 and CcSMC4, Figure S2: Immunoblot analysis of CcSMC4p antisense-knockdown and CcSMC2/4 antibodies, Figure S3: CcSMC4p-knockdown led to nuclear swelling in extended timepoints.
