# Supplementary material for: Knockdown of Dinoflagellate Condensin CcSMC4 Subunit Leads to S-Phase Impediment and Decompaction of Liquid Crystalline Chromosomes
Source: Microorganisms. 2020 Apr 14;8(4):565. doi: 10.3390/microorganisms8040565 (PMC7232253; doi:10.3390/microorganisms8040565)
Supplement: Supplementary file 1 [file microorganisms-08-00565-s001.pdf]

Supplementary Figure Legends

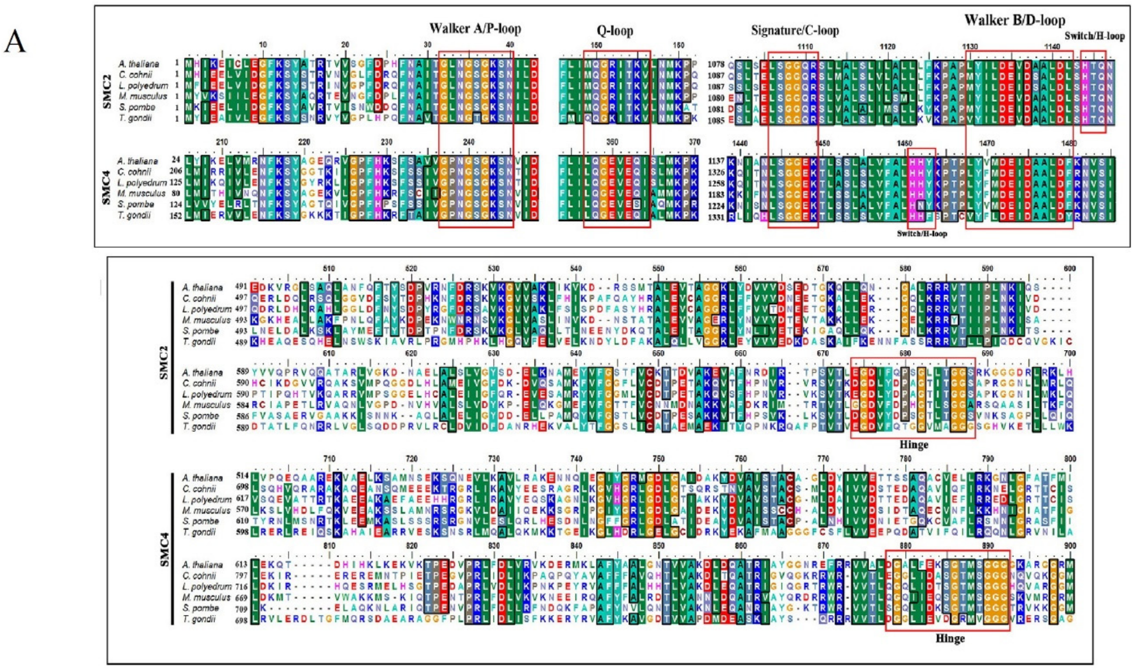

Figure S1. Predicted amino acid sequences of dinoflagellate CcSMC2 and CcSMC4. Sequence alignment between predicted selected domains of eukaryotic SMC2 and SMC4 with dinoflagellate homologues (*C. cohnii and L. polyedrum*): *Arabidopsis thaliana* (NP_201047.1, Q9FJL0.1*)*, *Schizosaccharomyces pombe* (NP_596180.1, NP_595392.1), *Toxoplasma gondii* (XP_002371200.1, AZ57432.1), *Mus musculus* (NP_032043.3, NP_598547.1). Conserved regions were shown and labeled: (A) the N-terminal and C-terminal Walker domains (ATPase); (B) the hinge domains at central region. Predicted CcSMC2p and CcSMC4p polypeptides have 30-40% sequence identity with other eukaryotic homologues.

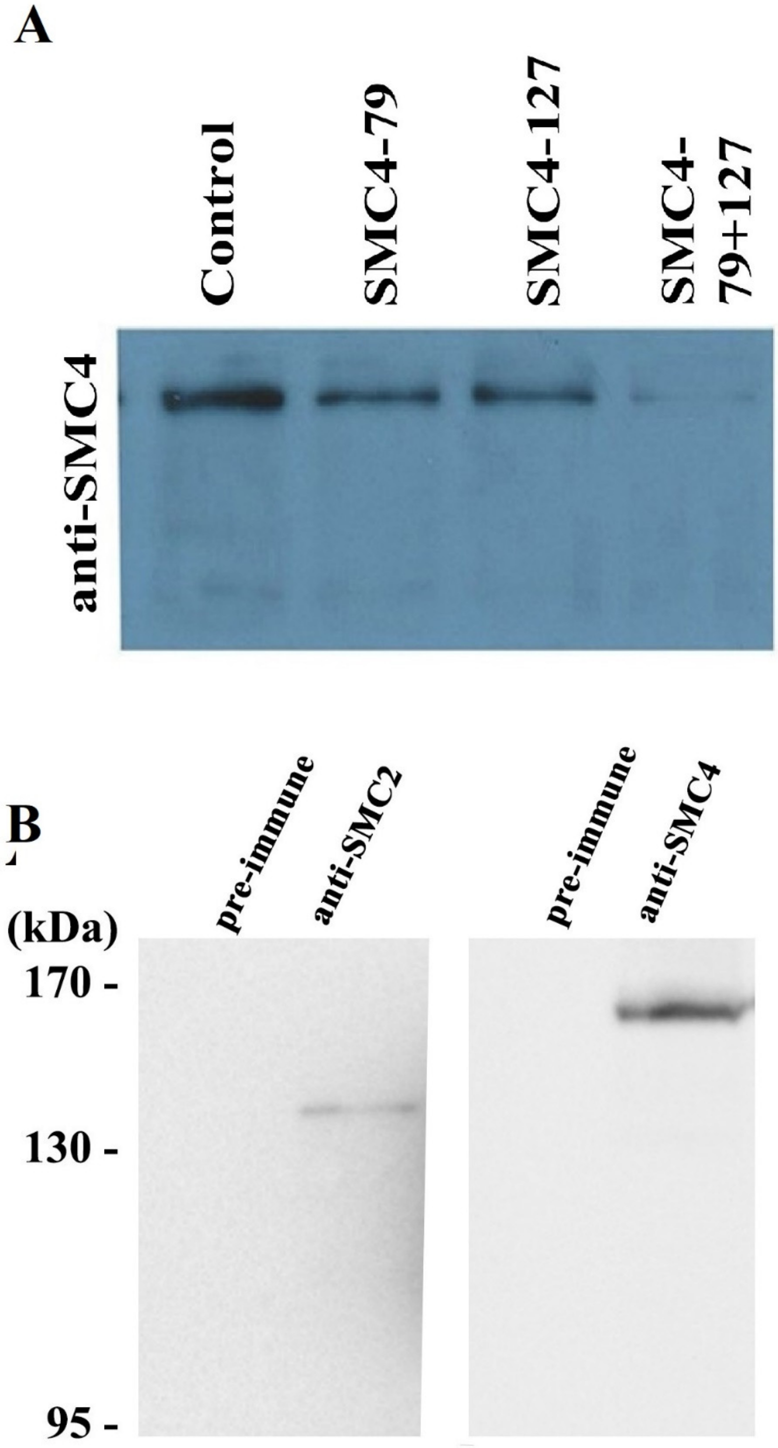

Figure S2. Western blot analysis of CcSMC4p antisense-knockdown and CcSMC2/4 antibodies. (A) Western blot of cell lysates prepared from cells with different antisense-ODNs (79, 127 or 79 + 127) on CcSMC4p level. Cell lysates were prepared from harvested cells at T=15 hr post-transfection. (B) Western blot of pre-cleared cell lysates with anti-CcSMC4p and anti-CcSMC2p antibodies; Positive bands approximately corresponded to predicted sizes of CcSMC2p (137 kD) and CcSMC4p (172 kD) were labeled. Affinity-purified antibodies were used.

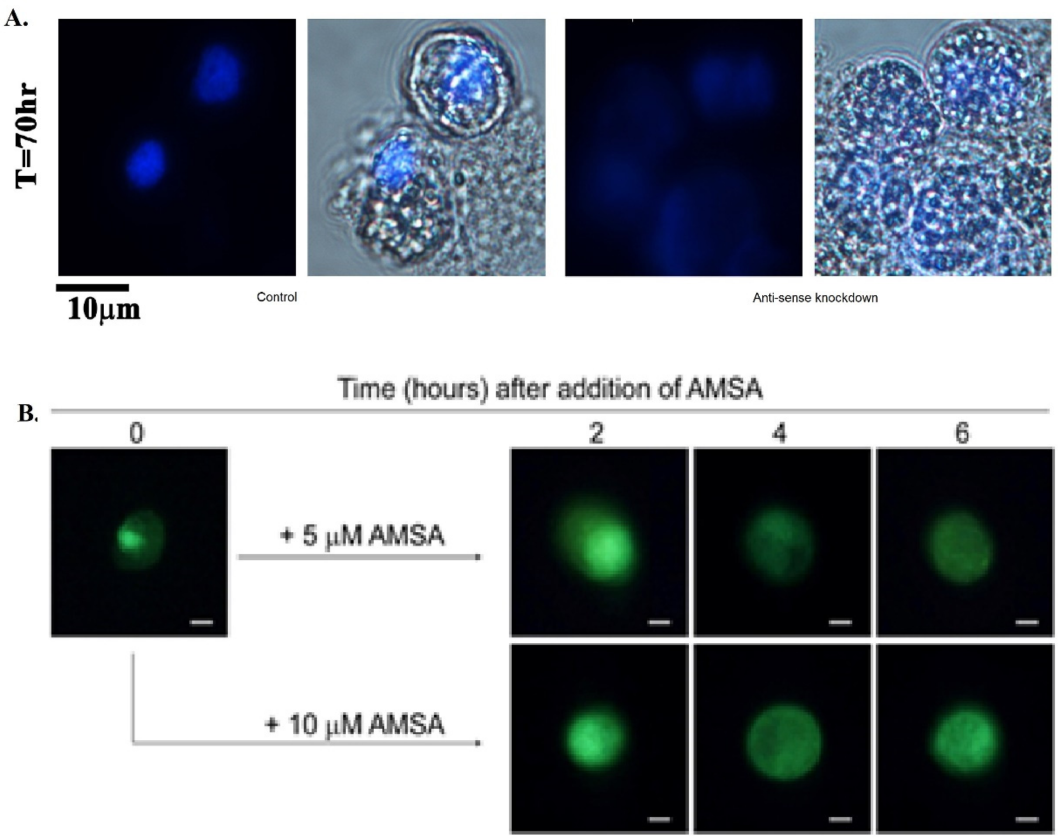

Figure S3. CcSMC4p-knockdown led to nuclear swelling at extended time points (A) DAPI staining suggested some ak-cells (T=70 hrs) had DNA staining covering the whole cells, similar EDTA-mediated nuclear swelling (B) or inhibition of topoisomerase II (Mak et al., 2007), instead of enlarged nuclei as in Figure 5. (B) Chelation-induced (15mM EDTA) LCC decompaction (Sun *et al*., 2012) led to nuclear swelling, with diffused DAPI-stained LCCs and associated CcSMC4p immune-stainings, contrasted with stainable LCCs in control *Karenia brevis* nuclei. (C)Topoisomerase II inhibitor AMSA dose-dependently led to nuclear enlargement in *C.cohnii* cells. Fluorescent photomicrographs of SYBR-Green I-stained *C. cohnii* cells that were treated with 5 or 10 μM AMSA and observed at the indicated time (hours). Scale bar = 10 μm.
